# Characterization of the starch-acting MaAmyB enzyme from *Microbacterium aurum* B8.A representing the novel subfamily GH13_42 with an unusual, multi-domain organization

**DOI:** 10.1038/srep36100

**Published:** 2016-11-03

**Authors:** Vincent Valk, Rachel M. van der Kaaij, Lubbert Dijkhuizen

**Affiliations:** 1 Microbial Physiology, Groningen Biomolecular Sciences and Biotechnology Institute (GBB), University of Groningen, The Netherlands; 2 Top Institute of Food and Nutrition (TIFN), Nieuwe Kanaal 9A, 6709 PA, Wageningen, The Netherlands

## Abstract

The bacterium *Microbacterium aurum* strain B8.A degrades granular starches, using the multi-domain MaAmyA α-amylase to initiate granule degradation through pore formation. This paper reports the characterization of the *M. aurum* B8.A MaAmyB enzyme, a second starch-acting enzyme with multiple FNIII and CBM25 domains. MaAmyB was characterized as an α-glucan 1,4-α-maltohexaosidase with the ability to subsequently hydrolyze maltohexaose to maltose through the release of glucose. MaAmyB also displays exo-activity with a double blocked PNPG7 substrate, releasing PNP. In *M. aurum* B8.A, MaAmyB may contribute to degradation of starch granules by rapidly hydrolyzing the helical and linear starch chains that become exposed after pore formation by MaAmyA. Bioinformatics analysis showed that MaAmyB represents a novel GH13 subfamily, designated GH13_42, currently with 165 members, all in Gram-positive soil dwelling bacteria, mostly *Streptomyces*. All members have an unusually large catalytic domain (AB-regions), due to three insertions compared to established α-amylases, and an aberrant C-region, which has only 30% identity to established GH13 C-regions. Most GH13_42 members have three N-terminal domains (2 CBM25 and 1 FNIII). This is unusual as starch binding domains are commonly found at the C-termini of α-amylases. The evolution of the multi-domain *M. aurum* B8.A MaAmyA and MaAmyB enzymes is discussed.

Many micro-organisms are able to use starch as a carbon and energy source. They generally employ α-amylase enzymes for extracellular hydrolysis of amylose and amylopectin^1^. α-Amylases represent endo-acting glycoside hydrolases that hydrolyze the (1–4)α-D-glucosidic linkages between glucose residues and rapidly degrade long polymers into shorter oligosaccharides. Degradation into glucose requires the subsequent action of exo-acting enzymes such as α-glucosidase or glucoamylase. Most α-amylases belong to the Glycoside Hydrolase family 13 (GH13) (www.CAZy.org)^2^. Based on sequence similarity, family GH13 enzymes are currently classified into 41 subfamilies^2^,^3^. So far α-amylases have been found in 15 of these subfamilies (GH13_1, 5, 6, 7, 10, 14, 15, 19, 24, 27, 28, 32, 36, 37, 39, 41)^4^,^5^. Not all GH13 α-amylases listed in the CAZy database have been classified into one of the currently established subfamilies^2^,^3^,^4^. α-Amylases have a catalytic domain that usually consists of 3 regions. Region-A contains the common (β/α)_8_ barrel and all the catalytic residues^6^. Region-B is a loop lacking a clearly defined topology, located between β3 and α3 of region-A, containing calcium binding sites^6^. Due to the close interactions between regions-A and -B they are usually identified as one (catalytic) domain in domain databases such as the Conserved Domain Database (CDD)^7^. Region-C is located at the C-terminal end of regions-AB. It consists of β-sheets only, including a Greek key motif^4^,^6^,^8^. Although it is required for amylase activity, the function of region-C is not yet fully understood^9^,^10^. In some cases region-C has been linked to raw starch binding^11^,^12^.

α-Amylase enzymes acting on raw starch commonly have additional carbohydrate binding modules (CBM) at their C-terminal ends^2^,^13^,^14^. Starch binding domains (SBD) are a subgroup of CBMs which bind to starches^14^. Only about 10% of all GH13 enzymes contain additional domains such as SBDs. Most do not have more than two of them, although some α-amylases contain several additional domains (6 or more) resulting in large complex enzymes^2^,^15^. We recently described MaAmyA, a large α-amylase (148 kDa) with two CBM25 and four FNIII domains, plus a novel CBM74 domain, that is able to form large pores in granular starch (Fig. 1)^15^,^16^. MaAmyA has been isolated from *Microbacterium aurum* B8.A, a granular starch degrading bacterium that was obtained from a potato waste water treatment plant^17^. Interestingly, the pores created by MaAmyA were all of similar size while the *M. aurum* culture fluid produced a range of pore sizes^16^. In addition, when staining for starch degradation activity on polyacrylamide gels, a 130 kD protein band was detected in the *M. aurum* culture fluid that could not be related to MaAmyA^16^. A total of 14 GH13 members were annotated in the recently obtained genome sequence of *M. aurum* B8.A (V. Valk, R. M. van der Kaaij, and L. Dijkhuizen, manuscript in preparation), one of which is located directly downstream of MaAmyA (designated MaAmyB). These findings, together with the reported synergy between α-amylases and glucoamylases which significantly increased the degradation rate of raw starches^18^,^19^,^20^,^21^, resulted in the suggestion that *M. aurum* B8.A may employ one or more additional enzymes to assist MaAmyA in raw starch degradation.

Here we report the characterization of MaAmyB, a large and multi-domain enzyme with exo-acting starch hydrolyzing activity (Fig. 1). MaAmyB is located directly downstream of MaAmyA in the genome of *M. aurum* B8.A (V. Valk, R. M. van der Kaaij and L. Dijkhuizen, unpublished data). Unlike the exo-acting glucoamylases, which belong to the GH15 family, MaAmyB belongs to the GH13 family and is the first characterized member of a newly defined GH13 subfamily (GH13_42). The 165 enzyme members of this subfamily share a conserved but aberrant multi-domain organization with additional domains N-terminal of their unusually large catalytic domain.

## Results

### Gene identification

**Figure 1:**
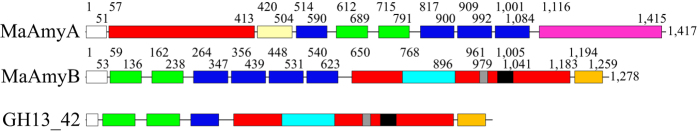
Detailed domain organization of MaAmyA and MaAmyB, compared to the general domain organization of GH13_42 members; numbers indicate the first and last aa of the domain or conserved insert. Domains are indicated as follows: white: signal sequence; red: AB-regions of the catalytic domain; pale yellow, C-region; blue: FNIII domain; green: CBM25 domain; pink: CBM74, a novel CBM domain^15^; orange yellow: aberrant C-region. Conserved insertions in the AB-regions of GH13_42 (including MaAmyB) are indicated as follows: light blue box: region-B; grey box: region-A insert 1; black box: region-A insert 2.

In previous work we characterized MaAmyA, a 148 kDa raw starch degrading and pore-forming α-amylase (glycoside hydrolase 13, subfamily 32: GH13_32) that was isolated from *M. aurum* B8.A (Fig. 1)^16^. The gene encoding MaAmyA was first isolated from a DNA library clone with an 11.6 kb insert. This insert contained a second large but C-terminally incomplete open reading frame (ORF) of 3,810 nt. The full 3,834 nt ORF was originally obtained through genome walking. The sequence was confirmed when the full genome of *M. aurum* B8.A was obtained (V. Valk, R. M. van der Kaaij and L. Dijkhuizen, manuscript in preparation). For this study the gene was obtained from genomic DNA of *M. aurum* B8.A using specific primers and designated *amyB*. It encoded a large multi-domain (putative) α-amylase of 1,287 amino acids, including a signal sequence of 53 aa, with a predicted mass of 135 kDa, designated MaAmyB. The genome sequence also confirmed that the gene encoding MaAmyB is located directly downstream of the gene encoding MaAmyA.

MaAmyB contains the A-, B-, and C-regions (but see specific details below) typical for the α-amylase superfamily^22^, as well as all catalytic residues and conserved regions generally present in family GH13 α-amylases^23^. MaAmyB does not belong to any established GH13 subfamily although a large number of sequences similar to MaAmyB was identified in databases (Table 1). We decided to heterologously express *amyB* and to study the activity and possible function of the produced *M. aurum* B8.A MaAmyB protein.

### Expression and purification of MaAmyB

**Figure 2:**
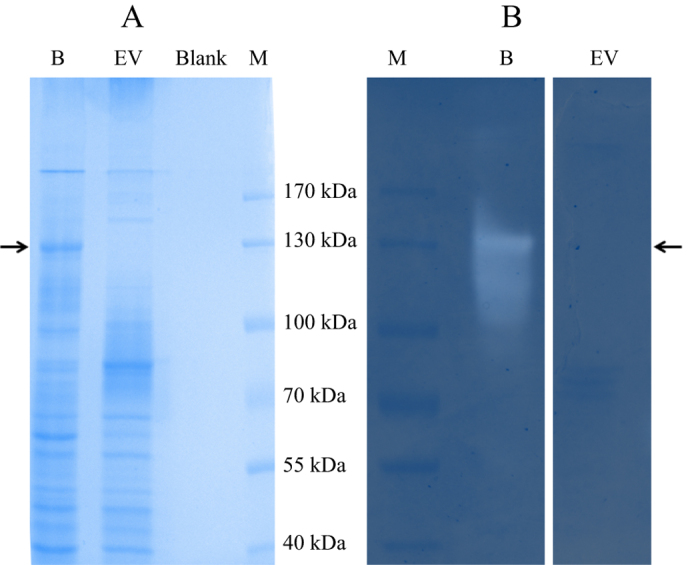
SDS-PAGE analysis and activity staining of MaAmyB. (**A**) Regular SDS-PAGE analysis. (**B**) In gel staining for starch hydrolyzing activity. Arrows indicate the position of MaAmyB. After SDS-PAGE the gels were washed with standard assay buffer, then incubated for 2 h at 37 °C in standard assay buffer containing 0.5% soluble starch and activity was visualized by staining with Lugol’s Iodine solution. The band seen in A corresponds to the main activity band in B. Both bands run at the predicted molar mass of 135 kDa for MaAmyB. The empty vector control did not show a band at 135 kDa (**A**) and lacked any other activity band (**B**). For each sample 10 μg protein was loaded. B = MaAmyB eluted from starch granules; EV = Empty vector control eluted from starch granules; Blank = Blank control eluted from starch granules; M = marker proteins.

MaAmyB was successfully expressed in *E. coli* as a full length protein with both N- and C-terminal His-Tags. During purification it became apparent that neither of the two His-Tags present was functional. Most likely they are not accessible in the folded protein structure. The MaAmyB enzyme was purified on basis of its efficient binding to wheat starch granules, followed by washing with standard assay buffer and elution with maltose. Purification was followed by SDS-PAGE analysis (Fig. 2). The MaAmyB starch hydrolyzing activity was confirmed through in gel iodine activity staining of the refolded MaAmyB protein incubated with soluble starch (Fig. 2B). A clear zone of activity with a visible band was apparent for MaAmyB while no activity was observed in the empty vector control sample, confirming the absence of *E. coli* starch degrading enzymes. The activity of MaAmyB was 62 ± 2 U∙μg^−1^ (CNP assay). MaAmyB activity was only observed in the presence of 10 mM of CaCl_2_.

### MaAmyB activity on different substrates

**Figure 3:**
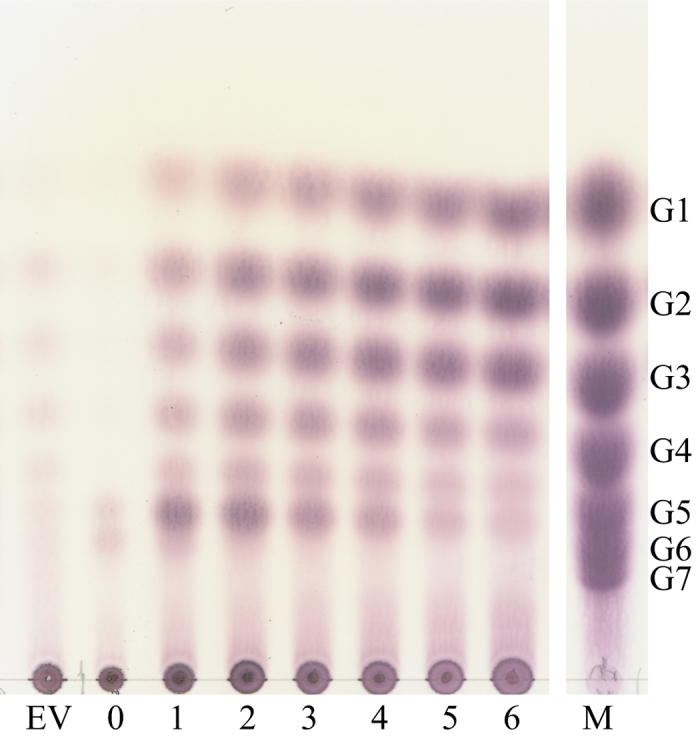
TLC analysis of MaAmyB products obtained in time by incubation of the enzyme (155 U, CNP assay) with soluble potato starch up to 6 h. EV = empty vector control incubated for 6 h. M = markers, size consisting of G1–G7 (glucose; maltose; maltotriose; maltotetraose; maltopentaose; maltohexaose; maltoheptaose).

MaAmyB was incubated with maltose (G2), maltoheptaose (G7) soluble and granular starch as substrates. The results showed that MaAmyB is inactive on G2, but active on G7 as well as on soluble and granular starches. Incubation of MaAmyB with soluble starch resulted in initial accumulation of maltohexaose (G6), which in time was further hydrolyzed to smaller maltooligosaccharides, resulting in accumulation of mostly maltotriose, maltose and glucose after 6 h (Fig. 3). Incubation with G7 yielded similar products, while incubation of granular starch with MaAmyB did not result in accumulation of maltohexaose but in direct release of glucose, maltose and maltotriose (see Supplementary Fig. S1).

**Figure 4:**
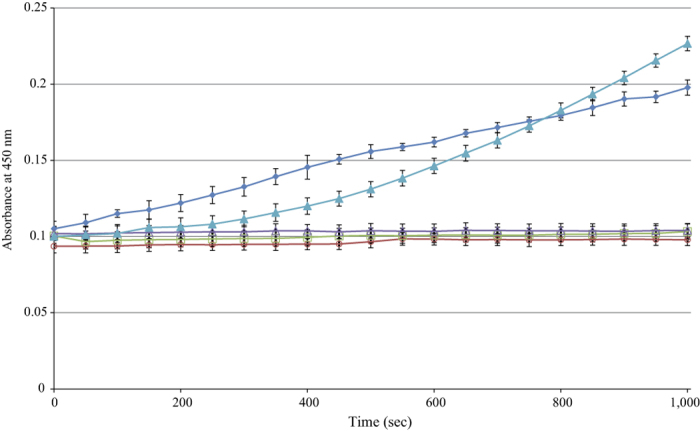
PNP release (absorbance at 405 nm) from the blocked PNPG7 substrate upon incubation with 6 U MaAmyB enzyme preparations and various control samples, (done in triplicate ± SD). Results obtained for MaAmyB and the positive control (EV + MaAmyA2 α-amylase + α-glucosidase) are significantly different (T-test for 0–500 sec, p-value: 0.028). 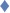 = MaAmyB; 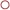 = empty vector (EV) control; 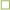 = Empty vector (EV) + MaAmyA2 α-amylase; 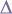 = EV + α-glucosidase; 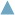 = EV + MaAmyA2 α-amylase + α-glucosidase.

MaAmyB activity was also determined using blocked PNPG7 as a substrate (Fig. 4). This substrate is generally used in a commercial kit for assaying α-amylase activity (Megazyme), based on the release of PNP. Normally the PNP is released in a two-step reaction, first involving endo-acting α-amylase activity to split the blocked substrate. In the second step, an exo-acting α-glucosidase activity (provided in large excess in the commercial kit) removes the remaining glucose units attached to the PNP group. When using limiting amounts of the exo-acting α-glucosidase the overall reaction shows an acceleration in time, due to the sequential action of the endo-acting α-amylase (PNPG7 cleavage) and the α-glucosidase (PNP release) (Fig. 4, blue triangles). To our surprise, MaAmyB alone was able to degrade blocked PNPG7 and showed immediate PNP release, resulting in straight lines of absorbance versus time (Fig. 4). Negative controls containing *E. coli* extracts prepared from cells with an empty vector (EV), or combined with either endo-acting α-amylase (MaAmyA2) or exo-acting α-glucosidase, showed no PNP release (Fig. 4). The combined data shows that MaAmyB acts as an α-glucan 1,4-α-maltohexaosidase, an amylase releasing a maltohexaose from soluble starch, also cleaving the blocked PNPG7 substrate, and thus is not affected by the 4,6-linked-O-benzylidine group at the non-reducing end of this substrate.

### Identification of MaAmyB as a member of a novel GH13 subfamily

**Figure 5:**
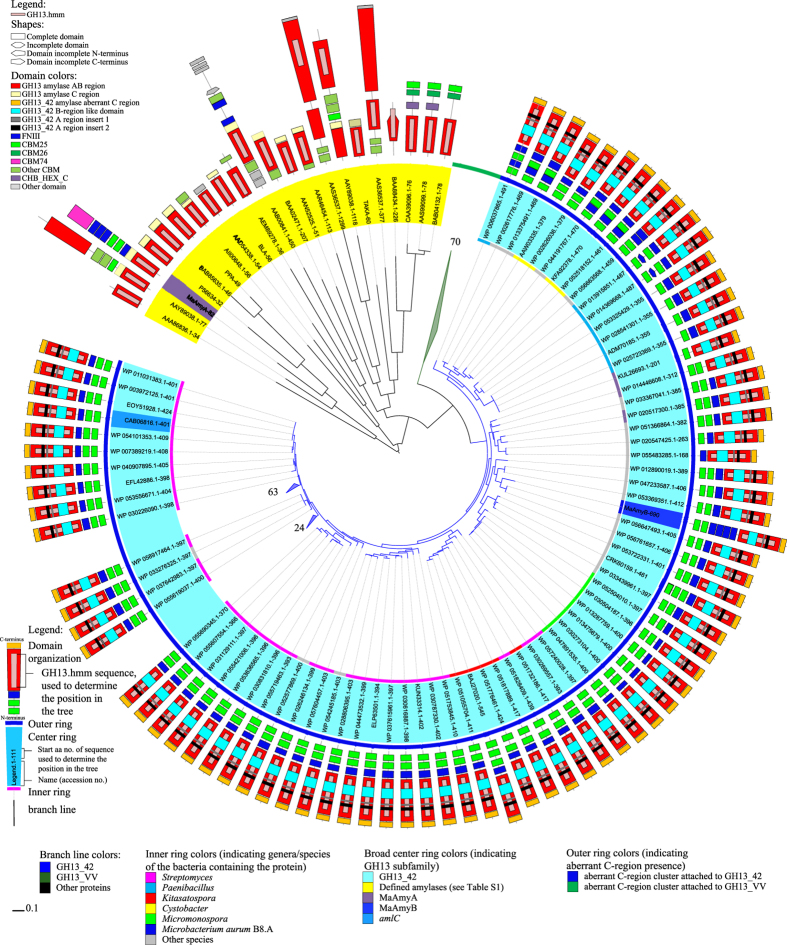
Phylogenetic tree of MaAmyB plus its 164 homologs, and a selection of members from the defined GH13 subfamilies that are either associated with EC3.2.1.1 or EC3.2.1.98^2^ (see Supplementary Table S1). The tree is based on the alignment of the catalytic AB-regions (GH13.hmm) as obtained from dbCAN. Domain organization shown is based on a combination of CDD and dbCAN data. The inserts in the A-region, the B-region and aberrant C-region as indicated are based on BLAST results of the identified regions from MaAmyB. Subfamily information was obtained from CAZy. Aberrant C-region information was obtained from Supplementary Fig. S2. The number 70 shows the location of a cluster of 70 putative α-amylases that share the aberrant C-region with the MaAmyB homologs but do not belong to subfamily GH13_42 (designated as GH13_VV) (see Supplementary Figs S2 and S3). At numbers 24 and 63, clades with these numbers of sequences similar to the nearby shown sequences have been collapsed to improve the readability of the tree. CBM74 is a novel CBM domain in MaAmyA^15^.

A search of the NCBI non redundant protein sequences database with the catalytic domain (regions-AB) of MaAmyB yielded a total of 164 sequences with at least 54% identity (February 2016). None of these putative starch hydrolases has been purified and characterized biochemically. Only AmlC of *Streptomyces lividans* TK24 has been identified as an α-amylase based on analysis in crude cell extracts (Yin *et al*. 1998). In the CAZy database, none of these MaAmyB homologs has been assigned to a specific GH13 subfamily. A phylogenetic tree containing all MaAmyB homologs, as well as selected enzymes from defined GH13 subfamilies, was prepared based on the partial AB-regions of the catalytic domain as identified by dbCAN (GH13.hmm in dbCAN), aa 690–1,121 for MaAmyB (Fig. 5). All MaAmyB homologs cluster together as a new group, which we propose to designate as the new GH13 subfamily 42 (GH13_42).

MaAmyB homologs were found predominantly in strains of the genera *Streptomyces* (115x), *Paenibacillus* (7x), *Kitasatospora* (7x), *Micromonospora* (6x), *Cystobacter* (4x). Up to three homologs were found in strains of 17 other genera. All GH13_42 homologs thus are found in soil-dwelling Gram-positive bacteria.

### GH13_42 domain organization: enlarged AB-regions and an aberrant C-region

**Figure 6:**
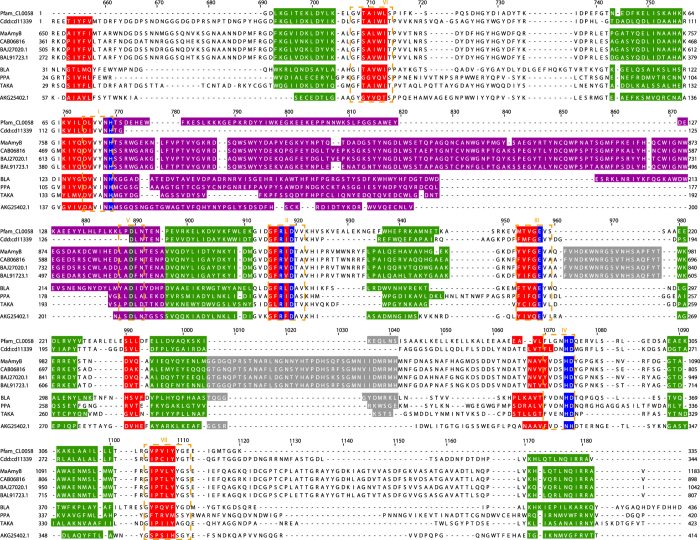
Alignment of the AB-regions of MaAmyB and 3 additional GH13_42 members (CAB06816, BAJ27020.1, BAL91723.1), Pfam CL0058 and CDD CD11339 from the databases and 4 characterized α-amylases: Pig pancreatic α-amylase (PPA) (P00690.3), Taka amylase A from *Aspergillus oryzae* (TAKA) (P0C1B4.1), *Bacillus licheniformis* α-amylase (BLA) (P06278.1) and MaAmyA (AKG25402.1). The B-region is indicated in purple, the two insertions in the A-region of GH13_42 are indicated in grey. The α-helices of the (β/α)_8_ barrel are indicated in green, β-sheets in red. Conserved Ca^2+^ binding aspartate residues are indicated in dark grey^23^. Other conserved residues are indicated in blue^23^. The 7 conserved regions of α-amylases are indicated with dashed orange lines and roman numbers^23^. Amino acid numbering as in MaAmyB.

**Figure 7:**
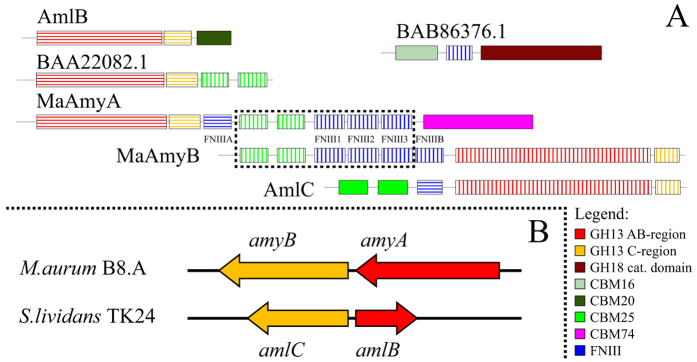
(**A**) Protein domain organization of MaAmyA and MaAmyB and 4 related proteins. All proteins are shown with the N-terminus on the left hand side. The dotted line box indicates a stretch of domains that is highly similar between MaAmyA and MaAmyB (>99% identity), suggesting a relatively recent duplication event. Equal fill patterns indicate domains with high similarity. Solid filled domains do not have similarity to any other domain shown. AmlB (CAB06622.1) and BAA22082.1 are included as representatives of GH13_32 with high similarity to MaAmyA. AmlC (CAB06816.1) is included as a representative of GH13_42 with high similarity to MaAmyB. BAB86376.1 is included as a representative of a group of chitinases containing FNIII domains with high similarity to the 3 C-terminal FNIII domains of MaAmyA and all FNIII domains of MaAmyB. CBM74 is a novel CBM domain^15^. (**B**) Schematic representation showing the orientations of the genes encoding the *M. aurum* B8.A (MaAmyA, MaAmyB) and *S. lividans* TK24 (AmlB, AmlC) α-amylases.

Most of the identified GH13_42 members share a similar domain organization starting with 2 CBM25 domains, 1 FNIII domain and the catalytic domain (AB-regions) (Fig. 5), which contains all catalytic residues and the 7 conserved regions generally found in the α-amylase superfamily (Fig. 6)^1^,^22^,^23^. The only exceptions are formed by two sequences separated by a single stop codon which are actually forming one single enzyme (CCK25026.1 and CCK25027.1), as well as one incomplete sequence (GenBank WP 03109885.1). Some homologs have an additional FNIII domain between the CBM25 domains. MaAmyB differs from its homologs in that it has 4 FNIII domains (Fig. 5), of which the N-terminal 3 are highly similar to each other (>97% identity), while the 4th FNIII domain has lower similarity (~55% identity) (Fig. 7).

In domain databases, α-amylases are listed as proteins with 2 domains: one domain represents the AB-regions, while the C-region is considered as a second and separate domain. The AB-regions of most GH13 α-amylases are represented by clan CL0058 in the Pfam conserved domain database^24^. In comparison to CL0058, the AB-regions of GH13_42 (534 aa for MaAmyB) are about 100 aa longer. The AB-regions of MaAmyB and 3 additional GH13_42 α-amylases were aligned with CL0058 from Pfam and 3 well-characterized α-amylases (Pig pancreatic α-amylase, Taka amylase A from *Aspergillus oryzae* and *Bacillus licheniformis* α-amylase) (Fig. 6). After manual tuning of the alignment using secondary structure information, 3 insertions could be identified in all GH13_42 members compared to CL0058 (Table 1). Two insertions were identified in the A-region. The first one is a 19 aa insertion between the 5^th^ β-sheet and 5^th^ α-helix (Fig. 6), which is uniquely found in all GH13_42 homologs (E value < 1∙10^−5^). The 2^nd^, a 37 aa insertion between the 6^th^ α-helix and the 7^th^ β-sheet, is predicted to form an α-helix located in close proximity to the C-region (Fig. 6). This insert is not only found in all 165 GH13_42 homologs but also in 41 additional putative α-amylases that share the aberrant C-region (see below and discussion) (E value < 1∙10^−4^). In addition, the B-region is 54 aa longer, compared to the 74 aa B-region from the CL0058 clan. This enlarged B-region is strongly conserved and uniquely found in GH13_42 homologs (Table 1).

The region C-terminal of the AB-regions in GH13_42 is not recognized by CDD as a classical C-region. Using Phyre2, which is based on protein (3D) structure prediction comparison, we observed a strong structural similarity (Phyre2 confidence of 97% or higher) between this C-terminal region in MaAmyB and several C-regions of α-amylases (such as the C-region of *Bacillus subtilis* α-amylase PDB1ua7, aa 348–425), although sequence identity was less than 30%. This low identity explains why the aberrant C-region of MaAmyB was not recognized by CDD. When used as a query sequence in a BLAST search, the aberrant C-region of MaAmyB (aa 1,194–1,259) returned 234 hits including all 164 GH13_42 members (query cover >95% and E-value < 1∙10^−13^), thus showing full conservation in the GH13_42 subfamily. The other 70 hits make up an additional cluster of putative α-amylases (designated as GH13_VV) which are not part of GH13_42 (see discussion). The aberrant C-region is 66 aa long, similar in length to known α-amylase C-regions. Phyre2 structure prediction for the aberrant C-region of MaAmyB showed an all β-sheet fold including a Greek key motif, as is typical for α-amylase C-regions.

### Evolutionary origin of MaAmyB

Figure 7 shows the domain organization of the *M. aurum* B8.A MaAmyA and MaAmyB proteins, and four closely related relatives. As representatives of the novel GH13_42 subfamily, MaAmyB of *M. aurum* B8.A and AmlC of *S. lividans* share the three inserts in the AB-regions (Figs 1 and 6) and the aberrant C-region (see Supplementary Fig. S2). The CBM25 and FNIII domains of MaAmyB however differ from those found in other members of GH13_42. The two CBM25 domains and 3 of the 4 FNIII domains of MaAmyB are almost identical (99% identity at the nucleotide level) to the domains found in the *M. aurum* B8.A MaAmyA (Fig. 7A). To study the origin of these duplicated domains, additional phylogenetic trees were constructed based on alignment of the CBM25 and FNIII domains (see Supplementary Figs S4 and S5). This revealed that the CBM25 domains of MaAmyB do not cluster with the same domains in the GH13_42 subfamily (26–45% identity) (represented by AmlC in Fig. 7A), but with CBM25 domains attached to 4 GH13_32 amylases, including MaAmyA (42–49% identity) (see Supplementary Fig. S4) (represented by BAA22082.1 in Fig. 7). Phylogenetic analysis also showed that the FNIII domains of MaAmyB and the 3 C-terminal FNIII domains of MaAmyA, do not cluster with the GH13_42 subfamily FNIII domains (18–38 identity, 32–59% similarity) but instead with FNIII domains found in chitinases (44–62% identity, 59–71% similarity) (represented by BAB86376.1 in Fig. 7A). The N-terminal FNIII domain of MaAmyA, however, clusters with FNIII domains of the GH13_42 subfamily (40–60% identity, 59–80% similarity) (see Supplementary Fig. S5). These results, combined with the fact that enzymes with a domain organization similar to those of MaAmyA and MaAmyB currently are not found in databases, suggest that the MaAmyA and MaAmyB enzymes originated after horizontal gene transfer and (intensive) gene (re)shuffling in *M. aurum* B8.A.

Interestingly, 15 of the 115 *Streptomyces* strains with a GH13_42 have a MaAmyA homolog located directly upstream of the GH13_42 homolog in their genomes. In these *Streptomyces* strains the GH13_42 is located on the complementary DNA strand, while the GH13_32 homolog is on the regular strand (Fig. 7B); the *M. aurum amy*A and *amy*B genes are both on the complementary strand. The *Streptomyces* MaAmyA homologs only possess a single CBM20 domain, lacking all of the CBM25, CBM74 and FNIII domains found in MaAmyA (Fig. 7A).

## Discussion

This paper reports the characterization of MaAmyB, a large and multi-domain amylase enzyme of *M. aurum* B8.A with hydrolytic activity on both granular and soluble starches, and on a blocked PNP-maltoheptaose. The highly unusual primary structure of this enzyme, with enlarged AB-regions as well as an aberrant C-region, was also identified in 164 other uncharacterized GH13 enzymes. Given the high similarity between these enzymes, we defined the new GH13_42 subfamily. MaAmyB is the only GH13_42 member with 4 FNIII domains, and with 2 CBM25 domains that do not cluster with the CBM25 domains in other GH13_42 members (see Supplementary Figs S4 and S5). Previously we reported that also the *M. aurum* B8.A MaAmyA α-amylase enzyme is unusual, the only known GH13_32 member with FNIII domains, and with a CBM74 domain that represents a novel SBD^15^,^16^. This raises questions about the evolutionary origins of these *M. aurum* B8.A MaAmyA and MaAmyB enzymes.

All currently know GH13_42 members share a general domain organization with 2 CBM25 domains and 1 FNIII domain N-terminal of the catalytic domain (Fig. 1). This strong conservation is remarkable as usually domain organizations vary within subfamily members^2^. Recently we have shown that CBM25 domains in MaAmyA are required for granular starch activity, but have no effect on soluble starch activity^16^. The role of FNIII domains in amylases has not been studied in detail. In MaAmyA truncation studies we did not see any effect for FNIII domains^16^. The role of FNIII domains in carbohydrate acting enzymes in general has not been studied in detail. Studies that addressed the roles of FNIII domains in such enzymes are inconclusive^25^,^26^,^27^,^28^,^29^,^30^,^31^,^32^,^33^,^34^,^35^,^36^,^37^. FNIII domains have been suggested to act as flexible linkers^25^,^29^,^36^ which corresponds to our findings for MaAmyA. CBM25 domains are usually located C-terminally of the catalytic domain, but N-terminally in GH13_42 proteins. We hypothesize that the FNIII domain functions as a flexible linker which enables the CBM25 domains in GH13_42 proteins to function efficiently at the N-terminus. With FNIII domains representing a linker to attach the CBM25 domains, and CBM25 domains enabeling enzymes to degrade granular substrates, we hypothesize that GH13_42 enzymes are involved in the degradation of granular starches.

The *M. aurum* B8.A MaAmyA and MaAmyB enzymes appear to have originated from at least three different enzymes: a GH13_32 α-amylase, a GH13_42 α-amylase and a chitinase (Fig. 7). Based on the sequence identities at the DNA level, we speculate that a single FNIII domain was duplicated twice resulting in three FNIII domains with high identities (95–98% at the nucleotide level). Together with 2 CBM25 domains, the 3 FNIII domains were duplicated between MaAmyA and MaAmyB. The high identity of the duplicated region (99% at the nucleotide level) suggests that this happened relatively recently. It is likely that in the environment from which *M. aurum* B8.A was isolated (a potato wastewater treatment facility), an efficient system for potato starch granule degradation is beneficial. We hypothesize that this evolutionary pressure caused horizontal gene transfer and gene (re)shuffling resulting in formation of MaAmyA and MaAmyB. This hypothesis is supported by the absence of enzymes with similar extensive domain organizations as MaAmyA and MaAmyB in the current databases, including 27 *Microbacterium* genome sequences^38^, (see Supplementary Figs S2, S4 and S5)^15^,^16^.

Members of the novel subfamily GH13_42 are characterized by enlarged AB-regions and an aberrant C-region (Figs 1 and 6). Compared to known α-amylases, the subfamily GH13_42 proteins have three strongly conserved insertions in the AB-regions (Table 1). The largest insert (54 aa) is found in the B-region which is known to be involved in calcium binding^6^. The aberrant C-region of GH13_42 proteins is strongly conserved among the subfamily members (Table 1). While it shares less than 30% sequence identity with the common C-region in GH13 α-amylases, it shows a strong structural similarity. Therefore it is likely that this domain functions as an α-amylase C-region. Additional bioinformatics analysis of 70 aberrant C-regions that were identified in family GH13 members that did not belong to the GH13_42 subfamily revealed that they are also part of putative starch hydrolases with enlarged AB-regions, though different from GH13_42. None of these 70 putative α-amylases have been characterized biochemically. A phylogenetic tree based on all 234 identified aberrant C-regions and some well know α-amylases shows that the aberrant C-regions cluster as a separate group away from other C-regions (see Supplementary Fig. S2). In addition, the 234 aberrant C-regions do not show clear clusters other than those expected based on the different species/genus harboring the genes encoding these proteins (see Supplementary Fig. S2). Despite the close sequence relationship between the aberrant C-regions, the additional 70 putative α-amylases do not belong to GH13_42 in view of the low sequence similarities in the AB-regions (<19% similarity); in a phylogenetic tree based on the AB-regions, they cluster together as an additional group, designated as GH13_VV (Fig. 5 and Supplementary Fig. S3). In these trees the various GH13_42 homologs cluster across multiple species, which is an indication for horizontal gene transfer of the AB-regions. Interestingly most of the GH13_VV members also have highly conserved, enlarged AB-regions with 3 inserts at similar places as in GH13_42. Region-A insert 2 is partly conserved between GH13_VV and GH13_42 (see Supplementary Table S3). Region-B and region-A insert 1 are uniquely conserved within the GH13_VV cluster (see Supplementary Table S3). Unlike GH13_42 not all regions are fully conserved in all GH13_VV members.

So far the aberrant C-region has only been identified in starch hydrolases with enlarged AB-regions that also have additional domains, like CBMs. In all GH13_42 enzymes and most GH13_VV members, additional domains are located N-terminal of the catalytic domain, while generally in GH13 such domains are located at the C-terminus of the enzyme^2^. The absence of domains attached to the aberrant C-region may be relevant (for its fold and function).

Structure prediction using the Phyre2 protein fold recognition server shows that region-A insert 2 in GH13_42 members forms an α-helix which lies in close proximity to the Greek key motif formed by the C-region (see Supplementary Fig. S6). Both the A-region inserts as well as the B- and C-regions in MaAmyB are predicted to be on the same side of the (β/α)_8_-barrel, although with low reliability (see Supplementary Fig. S6). This may indicate a functional relationship between the different enzyme parts as well as between the enlarged AB-regions and the C-region. We speculate that the C-region has a specific function in GH13_42, as may also be deduced from its concurrence with the enlarged AB-regions. Additional data preferably including the 3D structure of a GH13_42 homolog is needed to reveal the exact mechanism of interaction between the different domains, and the function of the C-region in GH13_42 enzymes.

Only one MaAmyB homolog, AmlC from *S. lividans* TK24, has been described previously^39^. AmlC is listed in CAZy as an α-amylase (EC 3.2.1.1), based on an activity analysis of crude extracts, which does not discriminate between α-amylases and exo-acting amylases. MaAmyB is able to release PNP from blocked PNPG7 without requiring any additional enzymes (Fig. 4). According to literature, the PNP from blocked PNPG7 can only be released through successive activity of an α-amylase and an exo-acting glycoside hydrolase enzyme, such as an α-glucosidase^40^. This is also demonstrated in one of the included controls, with MaAmyA2 α-amylase + α-glucosidase: due to the low exo-amylase activity used, an acceleration in the PNP release during the first 750 sec was observed (Fig. 4). Furthermore, α-amylases are not active on maltotriose or smaller substrates and release maltose as smallest product^41^. In this study, we show that MaAmyB can release glucose from small substrates (Fig. 3). It produces a series of oligosaccharides ranging from glucose to maltohexaose when soluble starch is used as a substrate. After 4 h the initially produced maltohexaose (G6) starts to decrease followed by degradation of maltopentaose (G5) and maltotetraose (G4). This pattern corresponds to that of an exo-acting enzyme with a preference for releasing G6 from starch, but which is also capable of producing glucose, like α-glucan 1,4-α-maltohexaosidases (EC 3.2.1.98)^42^,^43^,^44^,^45^,^46^,^47^. The activity of an α-glucan 1,4-α-maltohexaosidase on a blocked PNPG7 substrate has not been described in literature. Only the maltotetraose-forming amylase (EC 3.2.1.60) from *Pseudomonas saccharophila*^48^,^49^ is described in a GRAS notice^50^, as able to degrade blocked PNPG7 despite the blocking group, and to release maltotetraose (G4). It is likely that the maltohexaose releasing MaAmyB acts in a similar way (Figs 3 and 4). Contamination of MaAmyB by a low concentration of an *E. coli* α-amylase is unlikely as control experiments showed a clear increase in activity over time when an α-amylase and α-glucosidase were used (Fig. 4). In addition the empty vector control experiments did not show any activity even after prolonged incubation and addition of either an α-amylase or α-glucosidase. We therefore conclude that MaAmyB is an α-glucan 1,4-α-maltohexaosidase (EC 3.2.1.98).

The finding that MaAmyB acts as an exo-amylase allows for speculation about its function. Synergistic action between an α-amylase and glucoamylase in granular starch degradation has already been described^18^,^19^,^20^,^21^. MaAmyB is located in the proximity of an α-amylase (MaAmyA) on the *M. aurum* B8.A genome, and MaAmyA introduced a different pore pattern in starch granules than the *M. aurum* B8.A culture fluid^16^. MaAmyB also is active on granular starch (see Supplementary Fig. S1). Therefore we hypothesize that the exo-acting MaAmyB enzyme contributes to degradation of starch granules by rapidly hydrolyzing the helical and linear starch chains that become exposed after pore formation by MaAmyA. In addition, bioinformatics analysis revealed that in at least 15 *Streptomyces* species a (putative) GH13_32 α-amylase is located near a GH13_42 homolog. This suggests that the synergy between GH13_32 and GH13_42 members is more widely spread.

We conclude that MaAmyB is an α-glucan 1,4-α-maltohexaosidase, the first characterized member of the novel GH13_42 subfamily, which functions in the degradation of granular starch. It is likely that the enlarged AB-regions and aberrant C-region contribute to this specific activity. Further research into the structure/function relationship of these or similar enzymes with their N-terminal CBM domains, enlarged AB-regions and aberrant C-region, is required to elucidate the roles of their different protein domains.

## Materials and Methods

### Bacterial strains, media, and plasmids

*M. aurum* strain B8.A was isolated from a waste water treatment plant of a potato starch processing factory. Isolation and growth conditions have been described previously^17^. The *M. aurum* strain B8.A strain was deposited (deposit number LMG S-26033) in the BCCM/LMG culture collection of the University of Gent, Belgium^17^. *Escherichia coli* TOP10 and C43 were cultivated at 37 °C overnight in LB with orbital shaking (220 rpm). When required, ampicillin or kanamycin was added to a final concentration of 100 or 50 μg·ml^−1^, respectively. pBAD-VV^16^, a modified pBAD/Myc-His B (Novagen, Madison, WI, USA), was used as expression vector for the *amyB* gene constructs in *E. coli* C43(DE3).

### Gene identification

The full *amyB* gene was amplified from the *M. aurum* B8.A genome (V. Valk, R. M. van der Kaaij and L. Dijkhuizen, manuscript in preparation) Fwd and Rev primers: CGTCTTCGACCTCCATATGCGAAGGAGCAGGGTCTTGCGAGAAAGCACAC and GCCGGCACAGAGTGCTTGAGGTAGGCGCTGCCGCTACCGATCTTG. Chromosomal DNA was isolated from a *Microbacterium aurum* B8.A culture grown at 37 °C overnight in LB with orbital shaking (220 rpm) using the GenElute Bacterial Genomic DNA Kit (Sigma-Aldrich, Zwijndrecht, the Netherlands). The product was cloned into the pBAD-VV vector^16^ using NdeI and DraIII restriction sites. All products were confirmed through sequencing (GATC-Biotech, Konstanz, Germany). The *amyB* constructs were prepared with both N- and C-terminal His-Tags from the pBAD-VV vector.

### Protein expression

Recombinant *E. coli* strains were grown as 500 ml cultures in 3 l flasks, with 100 μg·ml^−1^ ampicillin and 0.05% (final concentration) arabinose (Sigma-Aldrich, Zwijndrecht, the Netherlands) for 6 h at 30 °C (220 rpm) and then for 40 h at 18 °C (220 rpm). Cells were collected by centrifugation at 4,250 *g* for 20 min at 4 °C (Thermo Lynx 4000). Pellets were resuspended in 50 mM Tris-HCl buffer pH 6.8 containing 10 mM CaCl_2_. Protease inhibitors (Roche Mini EDTA-free Protease Inhibitor, Sigma-Aldrich, Zwijndrecht, the Netherlands) were added and cells were broken by sonication (15 sec at 10,000 Ω, 30 sec cooling, 7x). Cell debris and intact cells were removed by centrifugation at 15,000 *g* for 20 min at 4 °C. Resulting cell free extracts were immediately used for purification of MaAmyB.

### Standard Assay buffer

A 50 mM Tris-HCl buffer pH 6.8 containing 10 mM CaCl_2_ was used as standard assay buffer for enzyme incubations. Unless indicated otherwise all incubations were performed at 37 °C.

### Purification of MaAmyB protein through binding to starch granules

Granular wheat starch (Sigma-Aldrich, Zwijndrecht, the Netherlands catalog no. S5127) was sterilized through gamma irradiation (Synergy Health, Ede, the Netherlands). The sterile granules were washed two times with standard assay buffer. After the second wash, granules were collected through centrifugation and the liquid removed. Then, 5 gram freshly washed (wet) granules was added to 20 ml of freshly prepared cell free extracts of the *E. coli* cultures expressing MaAmyB, or the *E. coli* empty vector culture as control. The tubes were mixed until a homogeneous suspension was obtained followed by incubating overnight at 4 °C on a roller bench (VWR, Amsterdam, the Netherlands). The suspension was centrifuged at 4,000 g for 5 min and the supernatant removed. The starch granules were washed 2 times with 40 ml standard assay buffer for 30 min, and the supernatant removed through centrifugation at 4,000 g for 5 min. To elute MaAmyB protein, 5 ml of 10% maltose in assay buffer was added, mixed for 30 min and the supernatant collected through centrifugation at 4,000 g for 5 min. The maltose was removed and the sample concentrated over 50 kD cut off Millipore spin filters and washed with standard assay buffer. Samples were concentrated to 1 ml and stored at 4 °C in standard assay buffer.

### Activity staining on SDS-PAGE

SDS-PAGE analysis was used to determine protein masses. After refolding the location of the MaAmyB protein could be visualized in the gels by staining for its starch-acting activity. SDS-PAGE^51^ was performed using precast THX gels (Bio Rad, Veenendaal, the Netherlands). After loading and running, gels were washed 3 times for 5 min in Milli Q water to remove SDS and allow protein refolding, then incubated for 2 h at 37 °C in standard assay buffer containing 0.5% soluble potato starch (Sigma-Aldrich, Zwijndrecht, the Netherlands)^52^. After incubation the gels were stained with Lugol’s iodine (2.5% I_2_/5% KI) to visualize protein bands with α-amylase activity. After imaging, gels were partly destained by washing in Milli Q water and then stained with Bio-Safe™ Coomassie Stain (Bio Rad, Veenendaal, the Netherlands) to visualize proteins. The Fermentas PageRuler prestained marker was included in each gel.

### Activity assay with CNPG3

The activity of MaAmyB and other enzymes was determined and standardized through incubation of the enzyme with 2-Chloro-4-nitrophenyl-α-D-maltotrioside (CNPG3). This compound has a 2-Chloro-4-nitrophenyl (CNP) group coupled to maltotriose (G3) at its reducing end. The assay is based on the detection of the released CNP group from CNPG3 by absorbance reading at 405 nm. The enzyme solution or mixture (15 μl) containing approximately 6.25 μg∙ml^−1^ MaAmyB protein to be tested was prepared in 96-well microtiter plates. Pre-warmed substrate (100 μl 2 mM CNPG3) was added and the reaction was followed through absorbance reading at 405 nm for 20 min in a microtiter plate reader (Spectramax plus, Molecular Devices, Sunnyvale CA, USA) set at 37 °C. One unit is defined as the amount of enzyme required to release 1 μmol CNP min^−1^ under standard assay conditions. This Unit definition is used throughout this paper.

### Activity assay with Blocked PNPG7 substrate

MaAmyB activity was tested by incubation of the enzyme with blocked 4-nitrophenyl-α-maltoheptaoside (blocked PNPG7) (Megazyme, Wicklow, Ierland). This compound has a 4-nitrophenyl (PNP) group coupled to maltoheptaose (G7) at its reducing end while the non-reducing end is blocked by an 4,6-linked-O-benzylidine group. Due to this block, release of PNP generally requires both endo-acting activity (α-amylase) and subsequently exo-acting activity (α-glucosidase). In the present work, blocked PNPG7 was tested as substrate by only adding the MaAmyB enzyme. The assay, based on the detection of the released PNP group from the blocked PNPG7 substrate by absorbance reading at 405 nm, was performed as described for the CNP assay using 6 U (based on the CNP assay) MaAmyB. Negative controls included an empty vector (EV) control without additional enzymes, EV with added α-glucosidase (Megazyme, Wicklow, Ierland), EV with added α-amylase (MaAmyA2, a truncation of MaAmyA from *M. aurum* B8.A with all starch binding domains removed^16^. A positive control consisted of EV with both α-glucosidase and α-amylase added. MaAmyA2 and the α-glucosidase were diluted in assay buffer until the activity on CNPG3 was 1.5–2 times the activity of MaAmyB on this substrate.

### Activity assays with other substrates

The activity of MaAmyB was tested on maltose (G2), maltoheptaose (G7), soluble potato starch (Sigma Aldrich, Zwijndrecht, the Netherlands, catalog no. S2004) and granular wheat starch (Sigma Aldrich, Zwijndrecht, the Netherlands, catalog no. S5127). The 900 μl substrate solution (or suspension in case of the granular wheat starch), containing 10 mg∙ml^−1^ of the substrate to be tested in standard assay buffer, was prepared and pre-heated at 37 °C in a heating block. Then 100 μl enzyme preparation containing 25 μg MaAmyB (155 U), or a control, was added. The solution was immediately mixed and the first 100 μl sample was taken and frozen (−20 °C) (t = 0 h). Additional samples were taken after 1, 2, 3, 4, 5, and 6 h for soluble substrates, and 0, 24, 48, 72, 96, and 120 h for granular substrates. Samples of 1 μl were analyzed on TLC (Merck, Darmstadt, Germany) and run overnight using a butanol, ethanol, water (5:5:3 v/v/v) running buffer, and stained using 40% sulfuric acid and 5% Orcinol (9,10-dihydrophenanthrene) in methanol^53^,^54^.

### Bioinformatics tools

All BLAST^55^ searches were performed with NCBI BLASTP using standard settings. The MaAmyB catalytic domain (aa 650–1,183) (regions-AB, Fig. 1) was used as query in BLAST searches for related sequences, using standard settings but with the maximum target sequences increased to 1,000 (instead of the default 100). Conserved domains were detected using both the NCBI conserved domain finder^7^ with forced live search, without low-complexity filter, using the conserved domain database (CDD), and dbCAN^56^ with standard settings. Signal sequences were predicted with SignalP4.1^57^ using standard settings. Catalytic AB-regions in the related GH13 proteins were identified using the full length sequences based on dbCAN domain information. The C-region in the related GH13 proteins was defined based on the BLAST search results obtained using the aberrant C-region of MaAmyB (aa 1,194–1,259) (Fig. 1). CBM25 domain sequences and FNIII domain sequences were extracted from all proteins listed in the 02–02–2016 release of CAZy^2^ (after their sequences were obtained from GenBank), based on CDD information. The extracted domain sequences were used for construction of alignments with Mega6.0^58^ using its build-in muscle alignment with standard settings. Alignments were checked for the positions of conserved residues and manually tuned when needed. Phylogenetic trees were made with Mega6.0 using the Maximum Likelihood method with gaps/missing data treatment set on “partial deletion” instead of “full deletion”. Trees were visualized with Interactive Tree Of Life v2^59^. The protein domain annotations shown in the trees are based on combined data from CDD and dbCAN. Information about GH13 subfamilies was obtained from the CAZy database^2^. An initial tree with a diverse selection of all GH13 subfamilies was constructed to select the closest related subfamilies for display in the final tree. The selection included multiple GH13 members from each of the 40 defined subfamilies, which varied in domain organization and origin. The final tree based on the catalytic domain regions-AB included MaAmyB and all homologs found in the NCBI non redundant protein sequences database, as well as a selection of members that were most closely related based on the initial tree. Protein structure predictions were done with the Phyre2 protein fold recognition server using standard settings^60^.

### Nucleotide sequence accession number

The sequence of *amyB*, isolated from *M. aurum* B8.A has been deposited into GenBank database under accession no. KX447523.1.

## Additional Information

**How to cite this article**: Valk, V. *et al*. Characterization of the starch-acting MaAmyB enzyme from *Microbacterium aurum* B8.A representing the novel subfamily GH13_42 with an unusual, multi-domain organization. *Sci. Rep.* **6**, 36100; doi: 10.1038/srep36100 (2016).

**Publisher’s note**: Springer Nature remains neutral with regard to jurisdictional claims in published maps and institutional affiliations.

## Supplementary Material

Supplementary Information

## Figures and Tables

**Table 1:** Overview of the conserved regions found in the catalytic domain of MaAmyB and their identity and similarity with either all identified homologs, or the GH13_42 homologs only.

Conserved region	Position in MaAmyB	Size	Sequences found	Identity	Similarity	E-value
Region-B	768–896	128 aa	164	48–79% (59%)	57–83% (68%)	<1∙10^−41^
Region-A, insert 1	961–979	19 aa	164	78–100% (86%)	83–100% (89%)	<1∙10^−5^
Region-A, insert 2	1,005–1,041	37 aa	205	44–81% (68%)	53–86% (73%)	<1∙10^−4^
C-region-like dom.	1,194–1,259	66 aa	234	32–75% (62%)	40–91% (81%)	< 1∙10^−13^
Region-A, insert 2	1,005–1,041	37 aa	164*	61–81% (71%)*	67–86% (75%)*	<1∙10^−6^
C-region-like dom.	1,194–1,259	66 aa	164*	52–75% (63%)*	75–91% (82%)*	<1∙10^−15^

The ranges are given for the percentage identity and similarity, as well as the average values, which are shown between brackets.

*values based on GH13_42 homologs only.
