# Supplementary material for: Characterization of the starch-acting MaAmyB enzyme from Microbacterium aurum B8.A representing the novel subfamily GH13_42 with an unusual, multi-domain organization
Source: Sci Rep. 2016 Nov 3;6:36100. doi: 10.1038/srep36100 (PMC5093618; doi:10.1038/srep36100)
Supplement: Supplementary Information [file srep36100-s1.pdf]

Characterization of the starch-acting MaAmyB enzyme from *Microbacterium aurum* B8.A representing the novel subfamily GH13_42 with an unusual, multi-domain organization

Vincent Valk[1,2], Rachel M. van der Kaaij[1], and Lubbert Dijkhuizen[1]*

[1]Microbial Physiology, Groningen Biomolecular Sciences and Biotechnology Institute (GBB), University of Groningen, The Netherlands
[2]Top Institute of Food and Nutrition (TIFN), Nieuwe Kanaal 9A, 6709 PA, Wageningen, The Netherlands

*Corresponding author: L.Dijkhuizen@rug.nl

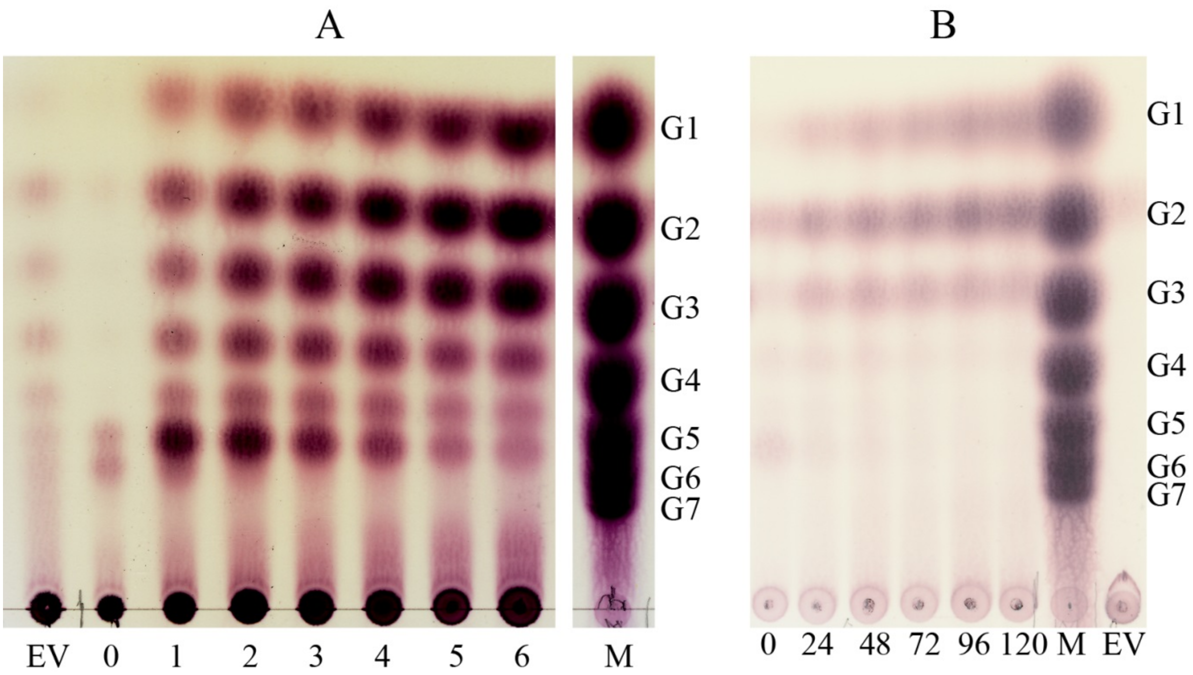

Figure S1. TLC analysis of MaAmyB products obtained in time by incubation of the enzyme (155 U, CNP assay) with maltoheptaose up to 6 h (A) or granular wheat starch up to 120 h (B). EV = empty vector control incubated for 6 h. M = markers, size consisting of G1-G7 (glucose; maltose; maltotriose; maltotetraose; maltopentaose; maltohexaose; maltoheptaose).

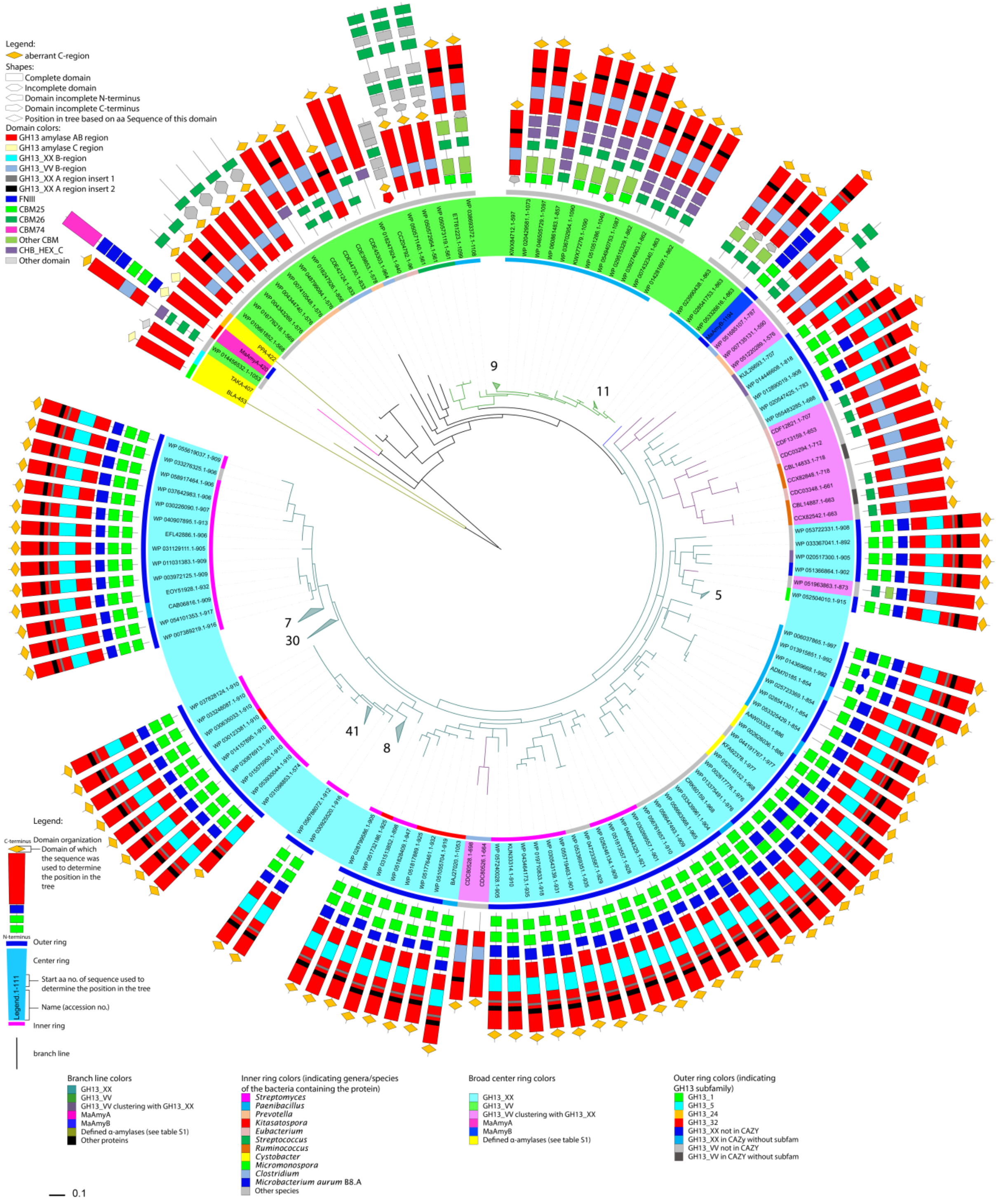

Figure S2. Phylogenetic tree of the 234 aberrant C-regions identified through a BLAST search with the C-region from MaAmyB (aa 1194-1259), three well characterized α-amylases: Pig pancreatic α-amylase (PPA) (P00690.3), Taka amylase A from *Aspergillus oryzae* (TAKA) (P0C1B4.1) and *Bacillus licheniformis* α-amylase (BLA) (P06278.1) as well as MaAmyA (AKG25402.1) which is directly upstream of MaAmyB in the genome of *M. aurum* B8.A. The tree is based on the alignment of the C-regions, indicated by a diamond shape in the domain organization as identified through BLAST searches. Domain organization within the protein is based on a combination of CDD, dbCAN data. The inserts in the AB-regions are indicated based on BLAST results of the identified regions from MaAmyB, as well as the identified B-region from CQR582041 for GH13_VV. Subfamily information was obtained from the CAZy database. Numbers indicate additional members which are similar to the nearby shown sequences, which have been collapsed to improve the readability of the figure.

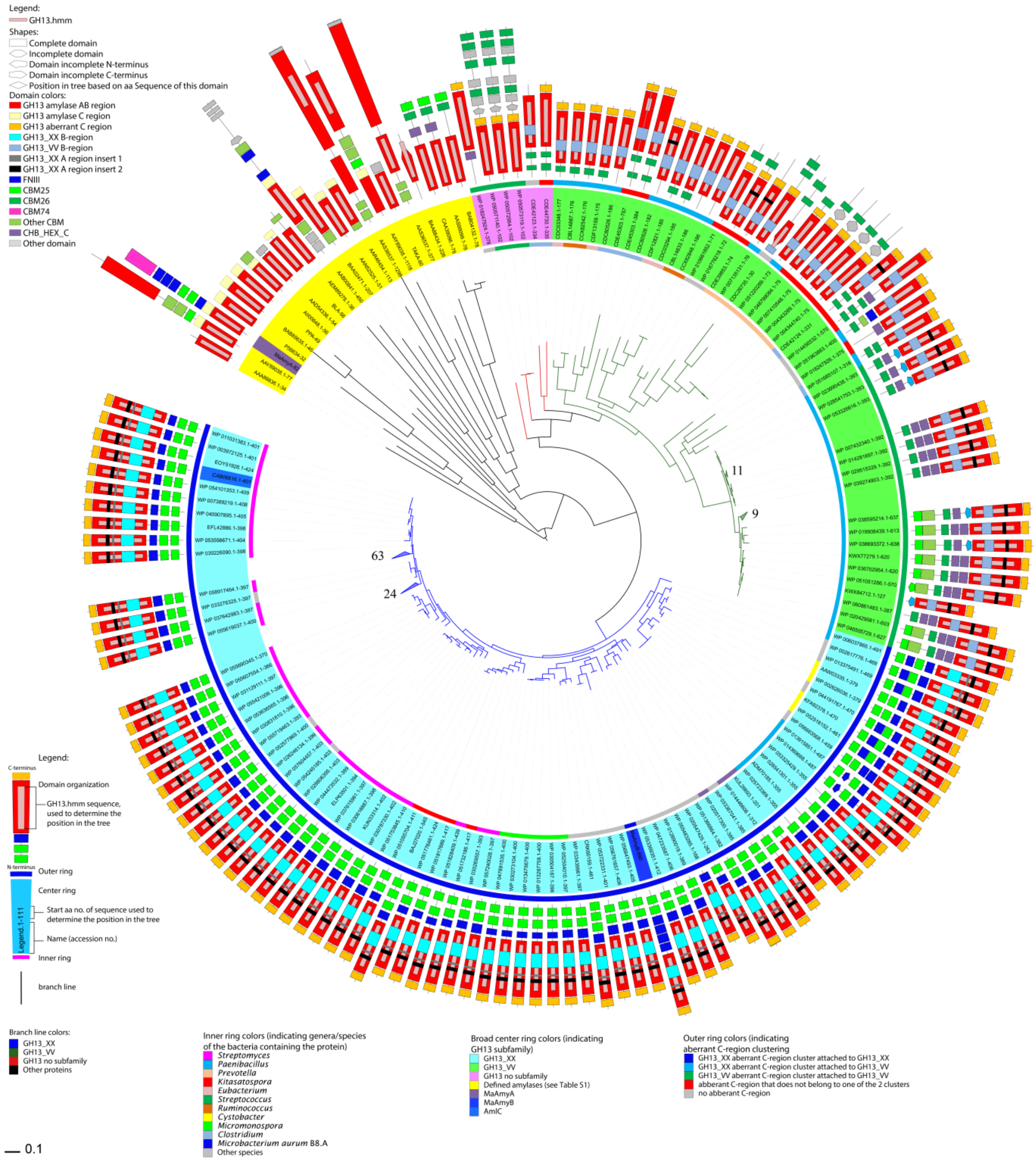

Figure S3. Phylogenetic tree based on the alignment of the catalytic AB-regions (GH13.hmm as obtained from dbCAN) of all 234 α-amylases with the aberrant C-region, 2 additional α-amylases with a GH13_VV B-region (CDE42123.1, CDC26735.1) and a selection of members from the defined GH13 subfamilies that are either associated with EC3.2.1.1 or EC3.2.1.98 [1] (Table S1). Domain organization within the protein is based on a combination of CDD and dbCAN data. The inserts in the A-, B- and C-regions are indicated based on BLAST results of the identified regions from MaAmyB (Table 1) and CQR58204.1 (Table S2). Species and (sub)family info was obtained from CAZy. Information about clustering of the aberrant C-regions was obtained from Figure S1. Numbers indicate clusters of additional sequences similar to the ones already shown, which have been collapsed to improve the readability of the tree. CBM74 is a novel CBM domain [2].

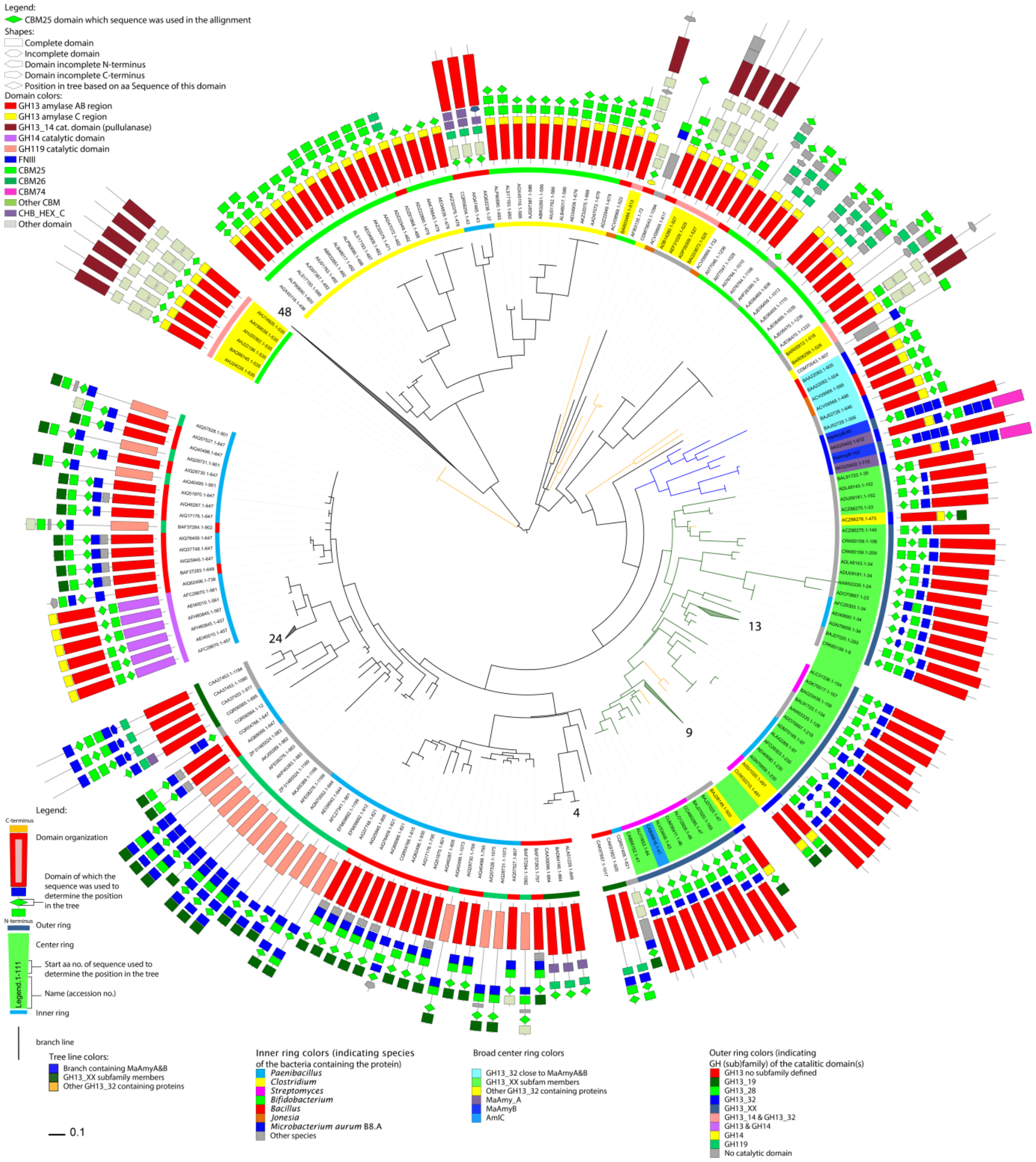

Figure S4. Phylogenetic tree of the 271 CBM25 domains identified by CDD, in proteins that contain CBM25 domains according to CAZy (which listed 275 CBM25 domains) as well as the 2 CBM25 domains from MaAmyB. MaAmyA (AKG25402.1) which is directly upstream of MaAmyB in the genome of *M. aurum* B8.A. is also indicated. The tree is based on an alignment of all CBM25 domains, indicated by a diamond shape in the domain organization. Sequences with multiple CBM25 domains are shown multiple times, once for each CBM25 domain. Domain organization within proteins is based on a combination of CDD and dbCAN data. Genes/species and subfamily information was obtained from the CAZy database. The number 48 indicates the 48 proteins that consist of only a CBM25 domain, not associated with any other protein domains. Other numbers indicate clusters of additional members which are similar to the nearby shown sequences, which are not shown to improve the readability of the tree. The branch containing MaAmyB is shown in blue on the broad ring, GH13_42 members are indicated by the broad green ring, and other proteins with a GH13_32 catalytic domain are shown in yellow.

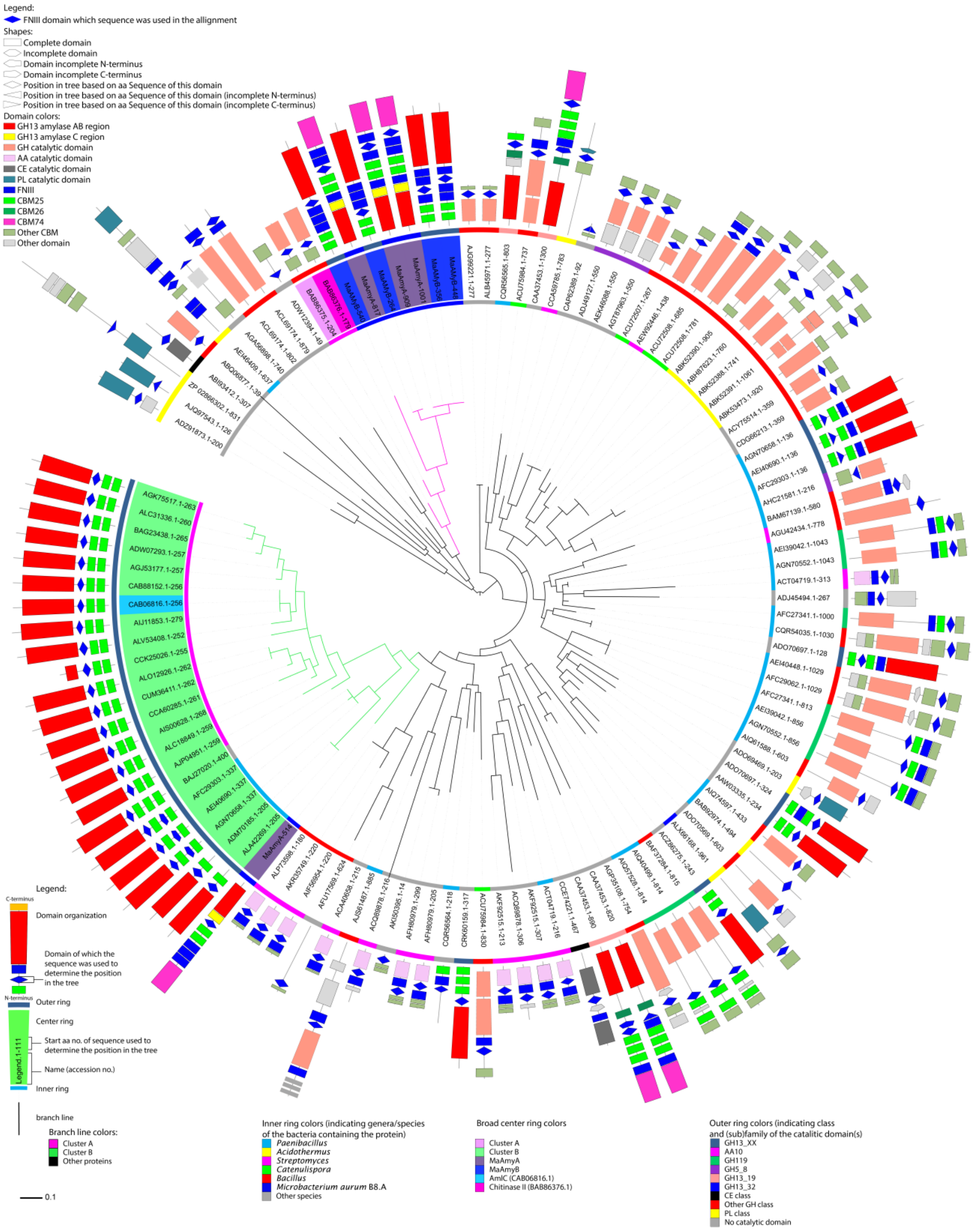

Figure S5. Phylogenetic tree of the FNIII domains identified by CD search (specific hits) in proteins that are listed in CAZy. The selection was based on an initial tree with all FNIII domains identified by CD search in proteins listed in CAZy. MaAmyB and MaAmyA (AKG25402.1), which is directly upstream of MaAmyB in the genome of *M. aurum* B8.A, are indicated. The position in the tree is based on the alignment of the FNIII domain indicated by a diamond shape in the domain organization. Sequences with multiple FNIII domains are shown multiple times, once for each FNIII domain. Domain organization within the proteins is based on a combination of CDD and dbCAN data. Enzyme class, family, subfamily and genus information was obtained from the CAZy database. The branch containing the first FNIII domain of MaAmyA is indicated as cluster A, the branch containing the remaining three FNIII domains of MaAmyA and all FNIII domains of MaAmyB is indicated as cluster B.

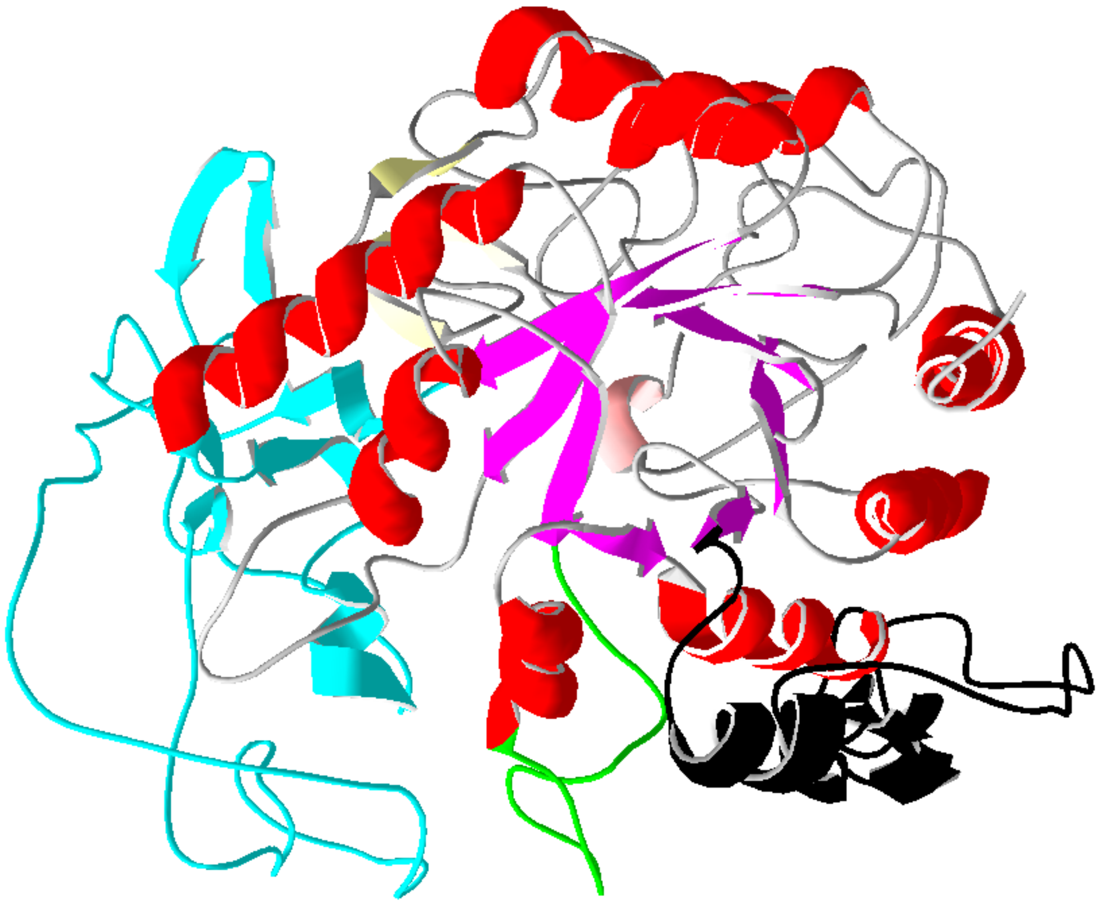

Figure S6. 3D model as predicted by the Phyre2 protein fold recognition server of MaAmyB without C-region. Although the model is less reliable for the exact structure of the inserts, it shows that all inserts are located next to each other, on the same side of the $(\beta/\alpha)_8$ barrel which may suggest a structural function. The C-region is not shown in the model but it is located on the right side directly above the region indicated in black. Blue = B-region; Green = region between β_5 and α_5 containing A-region insert 1; Black = region between α_6 and β_7 containing A-region insert 2; Red = α-helix part of $(\beta/\alpha)_8$ barrel; Pink = β-sheet part of $(\beta/\alpha)_8$ barrel; Pale red = α-helix not part of $(\beta/\alpha)_8$ barrel; Pale yellow = β-sheet not part of $(\beta/\alpha)_8$ barrel.

Table S1. Overview of well-known α-amylases used in the phylogenetic trees shown in Figure 5 and Figure S2. GH13 subfamily, EC number and Organism names were obtained from CAZy[1]. The members shown here were selected after an initial tree with a diverse selection of all (40) GH13 subfamilies was constructed to select the closest related subfamilies for display in the final tree. From these closest subfamilies, characterized members (if available with solved crystal structures) with α-amylase (EC 3.2.1.1) or α-glucan 1,4-α-maltohexaosidase activity were selected to display in the final tree.

Name in tree	GenBank accession no.	GH13 subfam.	aa no. of GH13.hmm	EC#	Organism name
AAA86836.1-34	AAA86836.1	27	34-352	3.2.1.1	*Pseudomonas* sp. KFCC10818
AAB00841.1-450	AAB00841.1	39	450-795	3.2.1.1, 3.2.1.41	*Thermoanaerobacterium thermosulfurigenes* EM1
AAD54338.1-54	AAD54338.1	7	54-341	3.2.1.1	*Pyrococcus woesei*
AAN52525.1-51	AAN52525.1	36	51-373	3.2.1.1	*Halothermothrix orenii* H 168 H 168; DSM 9562
AAR46454.1-113	AAR46454.1	10	113-424	3.2.1.1	*Sulfolobus solfataricus* KM1
AAS36537.1-377	AAS36537.1	none	1299-1639	3.2.1.1	*Bacillus* sp. KSM-1378
AAS36537.1-1299		12	377-691	3.2.1.41	
AAS99099.1-78	AAS99099.1	19	78-469	3.2.1.98	*Bacillus halodurans* LBK 34
AAY89038.1-77	AAY89038.1	32	1118-374	3.2.1.1	*Bifidobacterium breve* UCC2003
AAY89038.1-1118		14	77-374	3.2.1.41	
AEM89278.1-36	AEM89278.1	37	36-345	3.2.1.1	uncultured bacterium
AII00648.1-56	AII00648.1	6	56-340	3.2.1.98	*Corallococcus* sp. EGB
BAA02471.1-207	BAA02471.1	21	207-548	3.2.1.1	*Thermoactinomyces vulgaris* R-47
BAA88434.1-226	BAA88434.1	19	226-594	3.2.1.98	*Klebsiella pneumoniae*
BAB04132.1-78	BAB04132.1	19	78-469	3.2.1.98	*Bacillus halodurans* C-125
BAB85635.1-46	BAB85635.1	24	46-348	3.2.1.1	*Anguilla japonica*
BLA-56	P06278.1	5	56-403	3.2.1.1	*Bacillus licheniformis* MD1
CAA39096.1-76	CAA39096.1	19	76-467	3.2.1.98	*Bacillus* sp. H-167
CAB06816.1-401	CAB06816.1	XX	401-835	3.2.1.1/3.2.1.98*	*Streptomyces lividans* TK24
MaAmyA-82	AKG25402.1	32	82-370	3.2.1.1	*Microbacterium aurum* B8A
MaAmyB-690	XXXXX	XX	690-1121	3.2.1.98	*Microbacterium aurum* B8A
P56634-32	P56634	15	32-317	3.2.1.1	*Tenebrio molitor*
PPA-49	P00690.3	24	49-347	3.2.1.1	*Sus scrofa*
TAKA-60	P0C1B4.1	1	60-364	3.2.1.1	*Aspergillus oryzae* RIB40

*AmlC, is currently listed as an α-amylase (EC 3.2.1.1), but has not been characterized biochemically (see Discussion). Given the results found for MaAmyB as a member of this cluster, it is likely that also AmlC is actually an α-glucan 1,4-α-maltohexaosidase (EC 3.2.1.98).

Table S2. Overview of the conserved regions found in GH13_VV, α-amylases that have a similar aberrant C-region as found in GH13_42. The identity and similarity of these conserved regions are indicated for all homologs found, except those that belong to the GH13_42 family. The ranges are given for the identity and similarity, as well as the average values, which are shown between brackets. The positions are based on CQR58204.1, a predicted α-amylase from *Paenibacillus riograndensis* SBR5, which was used as model sequence for GH13_VV.

Conserved region name	Position in CQR58204.1	Insert size	Sequences found	Identity	Similarity
GH13_VV Region-B	705-798	94 aa	64	27-100% (70%)	34-100% (75%)
Region-A, insert 1	858-876	19 aa	65	28-100% (71%)	33-100% (74%)
Region-A, insert 2	910-942	33 aa	41*	67-100% (92%)	70-100% (93%)
Aberrant C-region	1073-1138	66 aa	70*	32-100% (76%)	38-100% (84%)

* = region also found in GH13_42 members.

Table S3. Overview of Stains, vectors and primers used during this study.

Host strain	*Microbacterium aurum* B8.A
Routine DNA manipulation strain	*E.coli* TOP 10
Expression strain	*E.coli* C43
Expression vector	pBAD-VV (see[3] for details)
MaAmyB primers:	
FW primer including DraIII restriction site (underlined)	GCCGGCACAGAGTGCTTGAGGTAGGCGC TGCCGCTACCGATCTTG
RV primer including NdeI restriction site (underlined)	CGTCTTCGACCTCCATATGCGAAGGAGC AGGGTCTTGCGAGAAAGCACAC
Sequence primer used for primer walking to find the last 24 nt of the initial (incomplete) MaAmyB gene	TCGGGCAACATGGCGTTCAAG

References:

1. Lombard, V., Golaconda Ramulu, H., Drula, E., Coutinho, P. M. & Henrissat, B. The carbohydrate-active enzymes database (CAZy) in 2013. *Nucleic Acids Res.* **42,** D490–D495 (2014).

2. Valk, V., Lammerts van Bueren, A., van der Kaaij, R. M. & Dijkhuizen, L. Carbohydrate Binding Module 74 is a novel starch binding domain associated with large and multi-domain α-amylase enzymes. *FEBS J.* **283,** 2354–2368 (2016).

3. Valk, V., Eeuwema, W., Sarian, F. D., van der Kaaij, R. M. & Dijkhuizen, L. Degradation of granular starch by the bacterium *Microbacterium aurum* strain B8.A involves a modular α-amylase enzyme system with FNIII and CBM25 domains. *Appl. Environ. Microbiol.* **81,** 6610–6620 (2015).
